# Review of Research Progress on Dry Granulation Technology for Blast Furnace Slag

**DOI:** 10.3390/ma18122802

**Published:** 2025-06-14

**Authors:** Hecheng Hu, Tuo Zhou, Ye Li, Bing Xia, Man Zhang, Nan Hu, Hairui Yang

**Affiliations:** 1 School of Energy and Power Engineering, Changchun Institute of Technology, Changchun 130012, China; 2 State Key Laboratory of Power Systems, Department of Energy and Power Engineering, Tsinghua University, Beijing 100084, China; 3 SINOSTEEL MECC, Beijing 100034, China

**Keywords:** slag treatment, dry granulation, centrifugal granulation, slag wool

## Abstract

Blast furnace slag, a high-temperature molten by-product generated during the ironmaking process in the metallurgical industry, has garnered significant attention for its resource utilization technologies. Compared to the traditional water-quenching method, dry granulation offers notable advantages. This paper systematically compares and analyzes the performance parameters of three typical dry treatment processes: mechanical crushing, air-quenching granulation, and centrifugal granulation. It reveals that the centrifugal granulation process demonstrates substantial technical superiority in key metrics, such as particle size distribution uniformity, particle morphology optimization, and heat recovery efficiency. Building on this, this study provides a comprehensive review of the current state of centrifugal granulation technology, from both experimental and simulation perspectives. Additionally, the combined processes of centrifugal granulation and air quenching can fully exploit the synergistic benefits of each technology, thereby enhancing overall efficiency. However, the wind’s cooling effect can lead to the premature solidification of molten slag when it splits into liquid filaments, resulting in slag wool. To address this, this paper proposes a centrifugal granulation device equipped with a windbreak board, which facilitates temperature zoning. This approach prevents premature solidification in the liquid filament region while ensuring the timely cooling and solidification of slag particles, offering a novel technical solution for optimizing centrifugal granulation in metallurgical solid waste resource utilization.

## 1. Introduction

Currently, China’s energy utilization remains plagued by issues such as low efficiency and poor economic returns. In the mid-to-late stages of industrialization, the industrial sector is the primary consumer of energy and a major source of pollutant emissions. Energy consumption in this sector accounts for approximately 70% of the nation’s total. The low utilization rate of industrial waste heat and the inadequate comprehensive use of energy are significant contributors to high energy consumption. China’s overall energy utilization rate stands at only about 33%, with at least 50% of industrial energy being discarded as various forms of waste heat. Waste heat resources constitute approximately 17% to 67% of the total fuel consumption, of which 60% is recoverable [1]. There is considerable room for enhancing waste heat utilization, which has vast potential for energy savings. The recovery and utilization of industrial waste heat is regarded as a “new energy” source and has become a crucial aspect of China’s energy conservation and emission reduction initiatives in recent years.

China has consistently held the top position in global steel production. According to data from the World Steel Association, the blast furnace pig iron output across 37 countries and regions reached 1.2865 billion tons in 2023, with China contributing 871 million tons, which accounts for more than half of the total production of the surveyed nations [2]. Blast furnace slag is the primary by-product of the blast furnace smelting process. Utilizing low-grade iron ore for ironmaking results in the production of 1.0 to 1.2 tons of blast furnace slag per ton of pig iron, whereas high-grade iron ore yields only 0.25 tons of slag per ton of pig iron [3,4]. The slag tapping temperature typically ranges from 1400 to 1600 °C, with one ton of slag containing approximately 1468 MJ of sensible heat, equivalent to the calorific value of 1.50798 million tons of standard coal, highlighting its significant potential for waste heat recovery [5,6].

The blast furnace slag treatment process has evolved over the past few decades, resulting in two primary methods: the wet and dry processes. In China, the overall utilization rate of blast furnace slag exceeds 90% [7]. The wet treatment of blast furnace slag is commonly employed in the current steel industry to produce water slag that serves as raw material for cement and concrete, thereby meeting the substantial demand of the cement sector. However, traditional wet processes are unable to achieve efficient waste heat recovery and suffer from drawbacks such as high freshwater consumption and the emission of harmful gases. In contrast, the dry granulation process offers significant advantages, including reduced water usage and the absence of harmful gas emissions, while ensuring efficient and stable heat recovery during the crushing of high-temperature molten slag. The development of dry granulation technology necessitates that, after waste heat recovery, the slag still meets the usage requirements for products like cement. This involves achieving a uniform granulation of blast furnace slag to produce high-vitrification rate products while also ensuring efficient and stable heat recovery. Nevertheless, this method currently faces challenges such as high energy consumption, insufficient kinetic energy for slag granulation, and a tendency to cause adhesion, which has hindered its widespread application in China.

To promote the industrial large-scale development of high-temperature slag waste heat recovery technology, this paper comprehensively reviews the current statuses and technical bottlenecks of dry granulation technologies across various scales in both domestic and international laboratories and industries. In response to the problem of increased slag wool production associated with the combined centrifugal and air-quenching technical route, a novel centrifugal granulation structure equipped with an additional windbreak panel is proposed. This innovative device effectively suppresses the formation of slag wool, thereby providing robust support for its widespread industrial application.

## 2. Dry Granulation Technology

Dry granulation technology primarily encompasses mechanical crushing, centrifugal granulation, and air-quenching granulation methods. The advantages of dry granulation include the following: it does not necessitate a significant amount of freshwater; the entire process recovers the sensible heat of blast furnace slag through direct or indirect contact with the heat transfer medium; and virtually no harmful gases are emitted during the process, thereby causing no environmental harm. The drawback is that for dry granulation to be competitive, both the operational costs and the quality of the slag particles must be on par with those of the water-quenching method. Undoubtedly, its potential environmental benefits should not be overlooked. In summary, this technology must fulfill the following five criteria:

(1) The subsequent heat can be recovered without causing secondary pollution.
(2) The energy loss of the blast furnace slag during the granulation process should be minimized.
(3) The energy consumption and operating costs of the granulation device should be kept as low as possible.
(4) The size of the granulated slag particles must be controllable to ensure product quality and effective waste heat recovery.
(5) Following heat recovery, the granulated slag particles should remain suitable for further effective use.

Research on the dry granulation process of blast furnace slag has been initiated both domestically and internationally, with the goal of directly recovering the high-quality latent heat from blast furnace slag. This recovered heat is then intended for use in power generation and the pyrolysis of biomass, among other efficient resource utilization methods [8].

### 2.1. Mechanical Crushing Technique

#### 2.1.1. Mechanical Stirring Method

**Figure 1:**
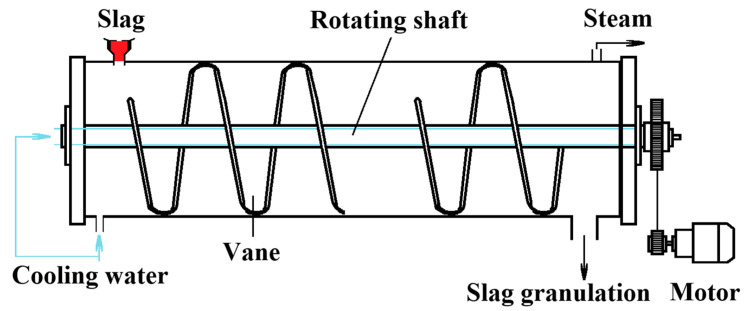
Mechanical stirring method [10].

In 1983, Sumitomo Metal Industries in Japan developed a mechanical stirring method for breaking down molten slag, as illustrated in Figure 1 [9]. This method employs rotating spiral blades to crush blast furnace slag. The drum’s outer wall is fitted with a water-cooled jacket to facilitate cooling and heat exchange during the crushing process. The resulting blast furnace slag particles can achieve a diameter of 2mm. However, the low cooling rate of the molten slag leads to a relatively low glass content, rendering it suitable only for use as road paving material. Furthermore, with a slag discharge temperature of approximately 900 °C, the method exhibits poor sensible heat recovery efficiency. Consequently, its application value is deemed limited, and subsequent scholarly research on this method has been virtually nonexistent.

#### 2.1.2. Continuous Casting and Rolling Process

**Figure 2:**
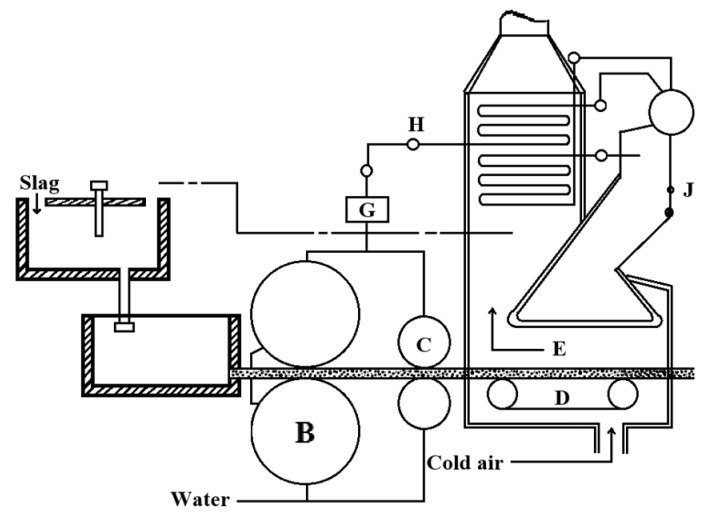
Continuous casting and rolling process [9].

The continuous casting and rolling method is derived from the metal continuous casting and rolling process. Chinese scholars adapted the slag dry granulation scheme developed by Dnepropetrovsk National Technical University, creating a dry rapid-cooling slag recovery system, as illustrated in Figure 2 [9]. However, the poor air permeability of high-temperature slag results in a low heat exchange efficiency within the system. The slag’s film-like structure necessitates additional energy for fragmentation. Furthermore, the design of the cooling and heat exchange section poses significant challenges, and the thermal recovery efficiency remains unvalidated, leading to a lack of reportable further progress.

Researchers at JFE Steel Corporation in Japan developed a novel plate-shaped steel slag-packed bed heat recovery process [11]. The study revealed that the modified Johnson–Rubesin equation, which incorporates a correction factor β, more accurately predicts the heat transfer coefficient of the plate-shaped slag-packed bed (β = 0.25–0.42), surpassing the traditional spherical particle model (Ranz–Marshall equation). During pilot tests, the countercurrent gas reached a temperature of 989 K after exchanging heat with high-temperature slag plates (1373 K) and achieved a heat recovery rate of 43%, with no fluidization observed in the slag bed. Further simulation analysis showed that increasing the packed bed height from 3m to 6m at an industrial scale could improve heat recovery efficiency (from 48% to higher) and decrease sensitivity to the β value. However, the process generated plate-shaped slag with large and uneven dimensions, presenting challenges due to its low economic benefits for reuse.

#### 2.1.3. Drum Granulation Technique

**Figure 3:**
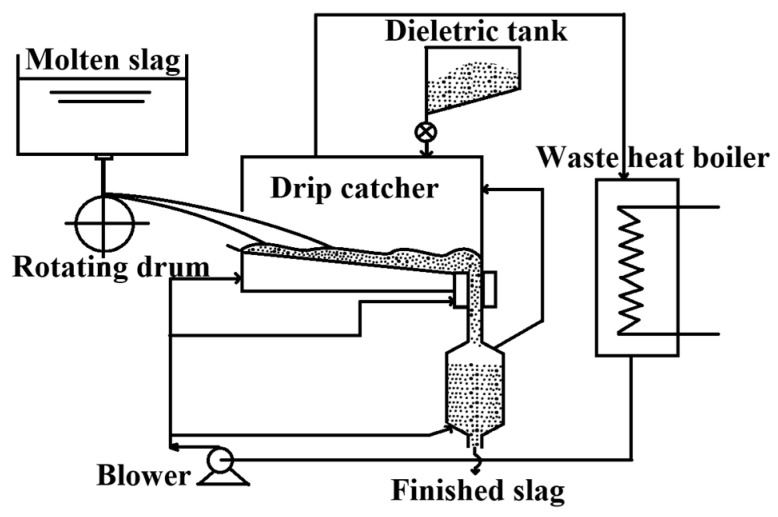
Single rotating drum [12].

In the late 1970s, Sumitomo Metal and Ishikawajima-Harima Heavy Industries in Japan jointly developed the rotary drum slag granulation process depicted in Figure 3 [12]. This process involves allowing molten slag to freely drop onto the surface of a rotating drum coated with a non-wetting material. The slag is then ejected from the drum and undergoes heat exchange with ambient slag particles and air, achieving granulation. Ultimately, it is sent to a fluidized bed along with the heat-exchanging slag particles for heat recovery. However, the selection of an appropriate coating for the rotary drum surface proved challenging, the separation of medium particles from high-temperature slag particles was difficult, and the heat recovery efficiency was low, which hindered its industrial application.

**Figure 4:**
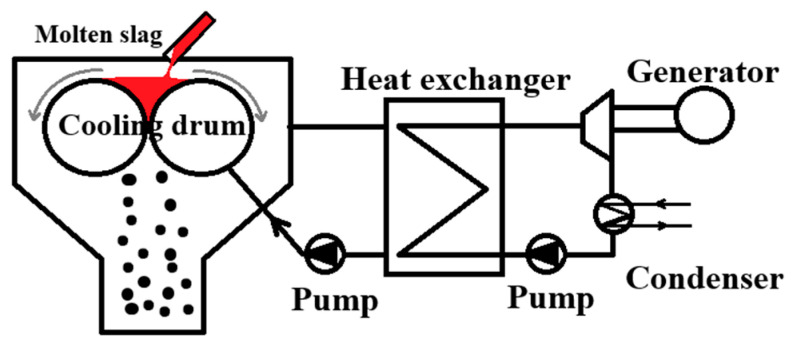
Twin rotating drum [13].

The double-drum method, illustrated in Figure 4, was subjected to industrial testing by Nippon Steel Corporation. Nevertheless, the slag discharge temperature remained high, the thermal recovery rate of the molten slag was below 40%, the machinery experienced significant wear, the equipment’s processing capacity was limited, and the produced slag predominantly exhibited a film-like morphology, which was detrimental to subsequent resource utilization. As a result, this process also failed to achieve industrial application [13]. Swiss researchers Bergkvist B. et al. [14] identified the challenges as being the design and stable operation of the drums; the control of slag film thickness; and the selection of organic solvents with high boiling points and high evaporation latent heat, which resulted in a low recovery rate that was unsuitable for practical applications.

### 2.2. Air Blast Granulation

#### 2.2.1. Air Blast Granulation at Industrial Scales

**Figure 5:**
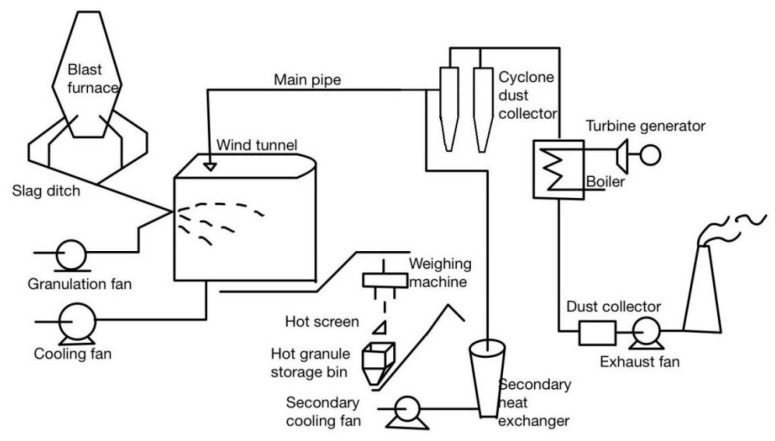
Air blast granulation method [15].

The air-quenching granulation method, also referred to as the wind-quenching method, primarily employs high-speed airflow to fragment blast furnace slag, thereby achieving granulation. This technique was initially jointly researched and developed by six major Japanese steel companies (Figure 5). Since 1982, it has undergone a total of six years of testing at the Nagoya Steel Works, where a semi-industrial wind-quenching steel slag granulation unit was established. This unit boasts a processing capacity of 40 tons per hour and achieves a heat recovery rate of over 65% [15,16,17,18,19].

During the same period, the Ma’anshan Iron and Steel Works also initiated research on this technology [20,21]. In 1993, China’s first blast furnace slag air-quenching equipment was established at the Chengdu Iron and Steel Works [22]. However, the actual energy consumption of this air-quenching system reached 48 kW·h/t, which was three times higher than that of the water-quenching process, leading to its shutdown due to poor economic viability. In 2017, the Shougang Group built the country’s first air-quenching demonstration line with an annual processing capacity of 500,000 tons. It featured a dual-stage swirl nozzle design that achieved an energy consumption of 1.2 kWh/t, marking a 40% reduction compared to traditional methods. By 2021, this system was upgraded to an integrated “air-quenching + fluidized bed” system [23].

In 2022, Baosteel Zhanjiang Iron and Steel Co., Ltd. developed a mobile air-quenching device [24], introducing a “pressure–flow rate” collaborative control algorithm. This innovation reduced compressed air consumption to 18 Nm^3^/t and addressed the issue of traditional fixed equipment’s inadequate adaptability to varying slag volumes. Chen Lixin and team [25] explored the quantitative relationship between air pressure and slag particle uniformity, ensuring the average slag particle size was maintained at 1.5 ± 0.3 mm, with a particle size standard deviation below 0.5 mm and a vitreous content of 94.5%. The energy consumption for slag treatment was lowered to 1.8 kWh, achieving 25% more energy savings compared to fixed equipment. Additionally, the mobile structure reduced the equipment maintenance cycle, boosting the annual operation rate to 91%. Industrial data reveal that this technology has reduced the comprehensive cost of blast furnace slag resource utilization by 18%, although further optimization of slag particle cooling uniformity is necessary to improve waste heat recovery efficiency.

#### 2.2.2. Laboratory Research on Air Blast Granulation

**Figure 6:**
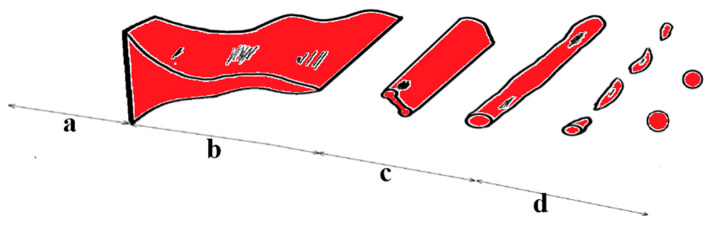
Molten blast furnace slag is quenched into beads [26].

Liu [26] studied the granulation mechanism of blast furnace slag air quenching through experiments and models. He explained the mechanism of the formation of pearl granules in blast furnace slag air quenching using wave theory (Figure 6). By optimizing the process conditions through steel slag modification, the blast furnace slag can be effectively air-quenched into glass microspheres.

Cai investigated the heat exchange characteristics of wind-quenched steel slag [27], with a focus on analyzing the flow field distribution within the granulation chamber and the effects of varying Mach numbers and nozzle diameters on this flow field. Increasing the Mach number can, to some extent, augment the impact kinetic energy imparted to the liquid slag, thereby enhancing the granulation process. Long Yue [28] also demonstrated through simulations that the granulation efficiency under 0.5 MPa nitrogen pressure surpasses that under 0.3 MPa.

In examining the impact of various parameters on the granulation performance of air-quenched blast furnace slag, Wang et al. [29] conducted numerical simulations of gas flow in a vacuum high-pressure air-quenching furnace to analyze its effect on the workpiece cooling process. Wang Lili [30] experimentally studied the effects of different airflow velocities and slag viscosities on the granulation performance of air-quenched blast furnace slag. It was observed that higher airflow velocities intensify unstable waves, causing liquid filaments to thin and become more susceptible to breakage. This, in turn, reduces the average droplet size while enhancing the heat exchange effect, which facilitates fiber formation. Zou and colleagues [31] investigated the wind-quenching granulation process and the impact of using stainless steel AOD slag as the raw material on the phase composition of the resulting products. They found that the influence patterns were similar to those reported by Zou et al. and noted that as the slag particle size decreases, the glass phase content progressively increases.

Regarding the flow field distribution within the granulation chamber, Wang Qiwu [32] discovered that high-speed jets exiting the nozzle generate a negative pressure zone in the surrounding gas medium. Excessive negative pressure results in a disordered distribution of slag particles, which hampers their efficient recovery. However, local negative pressure generates a velocity gradient in which the shear effect on liquid steel slag is more pronounced in higher-velocity areas, resulting in better granulation. The German SMS group [33] developed a modular air-quenching device (MAGMA^®^ system) to classify slag particle size and enhance resource utilization rates. Equipped with multi-stage cyclone separators, this system achieves particle size classification and increases resource utilization by 28%. Zhang Hongwei and colleagues from Northeastern University [34] developed an online particle size monitoring and feedback system based on machine vision. By integrating PID algorithms to adjust the gas–liquid ratio (GMR) in real time, they addressed the lag issue inherent in traditional manual sampling analysis.

Regarding the feasibility of employing wind-quenched slag as fine aggregate in the production of building materials like concrete, Chen Hongzhe and colleagues have investigated various fundamental properties. Wang Yan et al. [35] discovered that while its use in asphalt mixtures enhances water and low-temperature stability, it compromises high-temperature stability. As a blending material, wind-quenched steel slag exhibits poor grindability. A road constructed by the company using wind-quenched steel slag as a partial substitute for ordinary sand displayed varying degrees of bursting points and cracking within a decade [36].

The air-quenching method encounters several challenges in practical applications: high investment and maintenance costs for equipment; stringent control requirements for airflow velocity, temperature, and slag flow rate during the quenching process; and improper operation potentially resulting in uneven particle size or reduced activity, thereby impacting subsequent utilization efficiency. Moreover, the formation of fibers during air quenching can obstruct equipment, lower pelletization rates, and diminish the heat exchange efficiency between air and slag.

In conclusion, while the air-quenching method for treating molten slag offers notable benefits by enhancing resource utilization and mitigating environmental pollution, issues such as high cost, technical complexity, and adaptability constraints necessitate further research and refinement.

### 2.3. Dry Centrifugal Granulation

The centrifugal granulation method primarily fragments and granulates molten slag flow by utilizing the centrifugal force generated through mechanical motion. This method is mainly categorized into three types based on the granulator’s structure: the disk method, cup method, and drum method.

#### 2.3.1. Rotary Disk Atomizer

**Figure 7:**
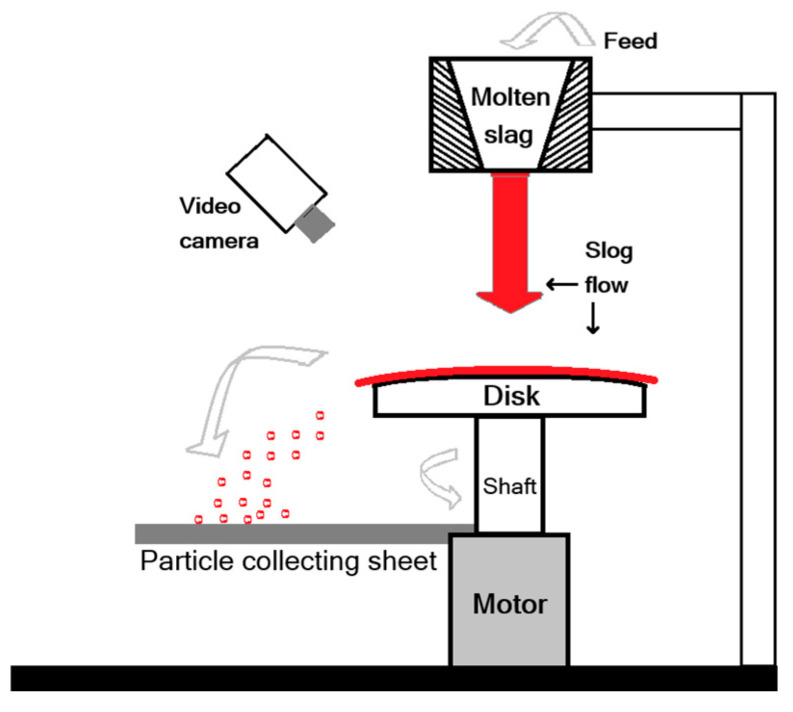
Rotary disk atomizer experimental apparatus [37].

In 1982, Yoshinaga et al. [37] pioneered granulation experiments on blast furnace slag using a rotating disk, observing that the particle size of the slag decreases as the initial temperature increases. They poured 1700 K molten slag onto a rotating disk granulator (Figure 7), capturing the particle formation process through photography. Furthermore, they developed a mathematical model, which was validated to align with experimental findings, to predict slag particle size and vitrification characteristics.

#### 2.3.2. Rotary Cup Atomizer

**Figure 8:**
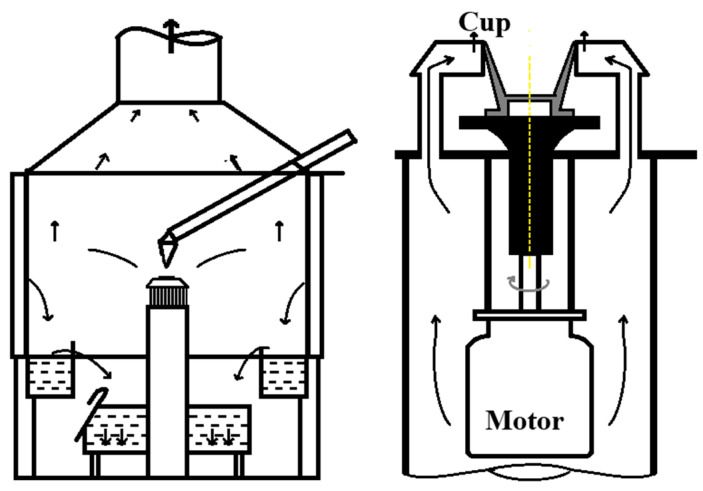
Blast furnace slag granulation experimental setup and rotating cup granulator structure [38].

The rotating cup method was introduced by Pickering et al. [38] in the mid-1980s. It was tested for blast furnace slag granulation at the British Steel Corporation (BSC) using a rotating cup pneumatic granulator, as depicted in Figure 8. An annular air jet was introduced around the rotating cup to create unstable fluctuations in the slag film, thereby enhancing slag fragmentation. Upon impacting the wall, the high-temperature slag particles fell into the primary fluidized bed for rapid cooling. These particles then overflowed into the secondary fluidized bed, allowing for additional heat recovery. Theoretical predictions suggest that this sensible heat recovery process could achieve a 60% efficiency rate upon industrial implementation.

#### 2.3.3. Rotary Cylinder Atomizer

**Figure 9:**
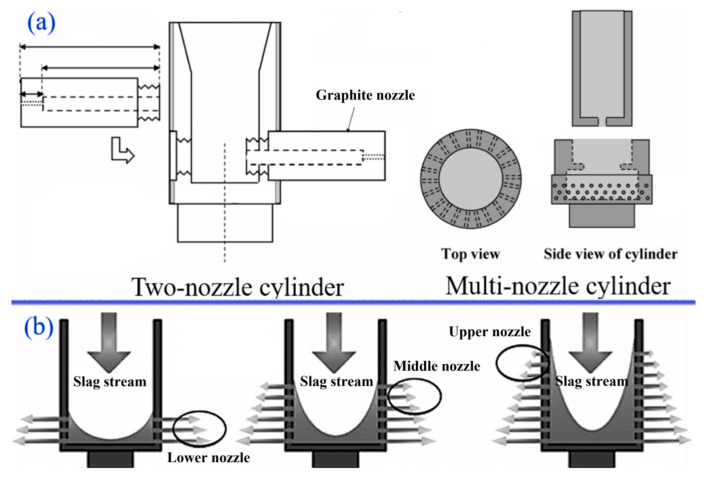
Rotary cylinder atomizer experimental apparatus [40,41,42,43].

The rotary cylinder atomizer was proposed by Kashiwaya et al. in 2010 [39] and led to the development of two types of rotary drum structures: double-nozzle and multi-nozzle, as depicted in Figure 9a. The minimum slag particle diameter ranges from 25% to 50% for the double-nozzle drum and from 10% to 50% for the multi-nozzle drum. This design enhances the sphericity of larger slag particles. Qin et al. [40,41,42] presented a rotary drum equipped with lower, middle, and upper nozzles, as depicted in Figure 9b. With a granulator nozzle diameter of 2 mm and a rotational speed of 1200 rpm, the slag particles measure less than 2.3 mm. Neuhold [43] also conducted relevant research on the rotary drum method, demonstrating that slag particle size can be directly controlled by the nozzle diameter. Despite its potential for controlling slag particle diameter via nozzle size, research on this method remains limited. In industrial production, it is essential to verify the occurrence of nozzle clogging in complex environments and during prolonged continuous operation.

### 2.4. Chapter Conclusion

Compared to the wet granulation process, the dry granulation method offers significant advantages. However, the uneven development of dry granulation technology and the lack of industrial implementation experience necessitate a comparative evaluation to select the optimal slag treatment technology. The aforementioned research reveals that mechanical stirring, continuous casting and rolling, and drum granulation all fall under mechanical granulation methods. Both mechanical stirring and continuous casting and rolling exhibit poor granulation effects and low residual heat recovery efficiency. Drum granulation leads to severe mechanical wear, complicating the application of surface coatings to the drum and resulting in uneven particle sizes of the crushed slag and low residual heat recovery rates, thus precluding its industrial application. The gas-quenching process demands precise control of airflow velocity, temperature, and slag flow rate; improper operation impairs subsequent utilization efficiency. Additionally, gas quenching readily forms fibers, which can clog equipment, reduce pelletization rates, and diminish gas–slag heat exchange efficiency.

Wu et al. [44] also employed a life cycle assessment method to compare the centrifugal granulation process with several common water-quenching processes, and confirmed the centrifugal granulation process’s superior performance in terms of energy consumption, environmental impact, and economics. However, the drum method’s susceptibility to clogging poses a critical issue at an industrial scale. Consequently, many scholars consider both the rotating cup centrifugal granulation method and the rotating disk centrifugal granulation method to hold significant research potential and promising development prospects. Therefore, the subsequent sections will concentrate on the development and prospects of centrifugal granulation.

## 3. Research on the Current Status of Centrifugal Granulation Technology

The centrifugal granulation method, deemed the most promising dry granulation technique, operates on the principle of using centrifugal force generated by a high-speed rotating granulator to break molten slag into particles. This method enjoys a superior position due to its notable advantages, including a simple structure, ease of operation, high amorphous rate, and low energy consumption. However, large-scale industrialization remains challenging, with issues such as granulator durability, slag particle uniformity, productivity, and efficient waste heat recovery. It is crucial to explore the underlying factors impeding the advancement of centrifugal granulation technology and to clarify the directions for its improvement and development. Consequently, numerous scholars have conducted laboratory-scale simulation studies and utilized commercial software for numerical simulations to investigate the flight, solidification, and heat exchange processes during centrifugal granulation.

### 3.1. Factors Influencing Granulation Effect in Experimental Studies

The centrifugal granulation process of blast furnace slag is highly complex and influenced by numerous factors. Scholars primarily concentrate their research on the operational conditions of the process, including the flow rate of blast furnace slag and the speed of the granulator; the composition and initial temperature of the slag; and the structural parameters of the granulator, such as the inner and outer inclination angles, depth, diameter, and roughness.

#### 3.1.1. The Influence of Various Experimental Conditions

The temperature of blast furnace slag can reach as high as 1400 °C, imposing stringent requirements on the design and construction of experimental setups during the laboratory research phase, as well as posing significant operational risks. Consequently, some scholars opt to use media with properties similar to slag for simulation experiments.

Xie et al. [45] employed tin powder to simulate the effects of process conditions on particle size and morphology during centrifugal granulation. Their experiments revealed that the granulator design, rotational speed, and melt flow rate significantly influence particle morphology and size distribution. They also identified liquid film thickness and wetting behavior as critical factors affecting granulation efficiency. Yu Qingbo et al. [46,47] used liquid paraffin as a slag substitute and found that slag temperature has no significant impact on particle diameter and mass distribution; however, increasing both the rotational speed and mass flow rate enhances particle mass distribution uniformity.

Nevertheless, paraffin’s similarity to blast furnace slag is limited to its melting temperature characteristics, making it less convincing for accurate simulation. Therefore, Min Yi and colleagues from Northeast University [48] developed a physical model based on liquid–solid transformation similarity, geometric similarity, and dynamic similarity. They proposed that the Reynolds number (Re), Weber number (We), and Ohnesorge number (Oh) between the simulation and actual conditions should be equal. When the geometric similarity ratio is 1:1, Oh depends solely on the working medium’s density, viscosity, and surface tension, indicating liquid–solid transformation similarity. A rosin–paraffin mass ratio of 4:1 meets both liquid–solid transformation and dynamic similarity criteria, providing robust support for subsequent researchers in selecting simulant materials for centrifugal granulation technology.

Building on Min’s findings, Dhirhi, from India [49], also utilized a 4:1 rosin–paraffin mixture to simulate blast furnace slag in conducting experiments to analyze the effects of rotational speed, disk diameter, and liquid flow rate on particle size. Dimensionless correlation equations (Re, Oh, and We) were established to predict particle diameter, and the critical operating conditions to avoid fiber formation were determined. These correlation equations were validated using laboratory data, leading to the proposal of a design method for industrial-scale slag granulation devices. The applicable ranges of flow rate and rotational speed were clarified, providing a theoretical basis for the recovery of waste heat from blast furnace slag and the production of cement substitute materials.

Fu et al. [50] focused on the granulation characteristics of molten slag under high flow conditions and the impact of different components on the granulation performance of blast furnace slag. Experiments revealed that under high flow conditions, molten slag undergoes film-like splitting, and increasing the granulator’s rotational speed can reduce particle size. Additionally, when blast furnace slag has a low MgO content or a high TiO_2_ content, its viscosity increases, resulting in a higher proportion of slag formation.

#### 3.1.2. The Influence of Various Granulator Structures

##### Typical Granulating Machine

**Figure 10:**
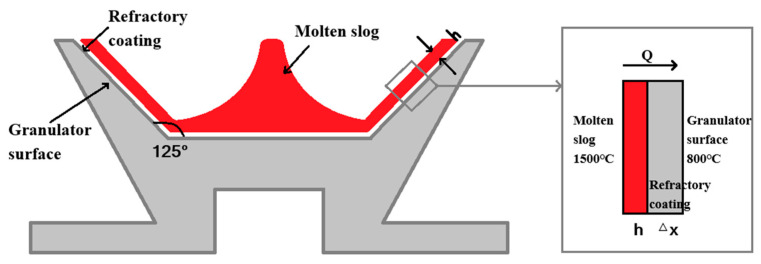
Surface heat exchange diagram of the mill coated with refractory material [45].

In Section 3.1.1, it was demonstrated that the diameter and design of the granulator (e.g., cup-shaped versus flat disc-shaped) are pivotal in determining particle size and morphology. Xie et al. [45] highlight that cup-shaped granulators, particularly those with high angles, significantly diminish particle size. Furthermore, Fu Yuxiang et al. [50] investigated the effects of refractory cement coatings on granulator thermal protection (Figure 10), revealing that these coatings reduce granulator temperatures and particle diameters, although excessively thick coatings elevate the slag formation ratio.

Regarding the surface roughness of granulators, Chang [51] and Zhang [52] et al. discovered that the particle size of molten slag is inversely proportional to the granulator’s surface roughness. The finer the slag particles become, the greater the frictional force applied to the slag. Chen [53] conducted an extensive study on disc granulators made from various materials and with different surface roughness levels, yielding results that aligned with these findings.

##### Atypical Granulating Machine

**Figure 11:**
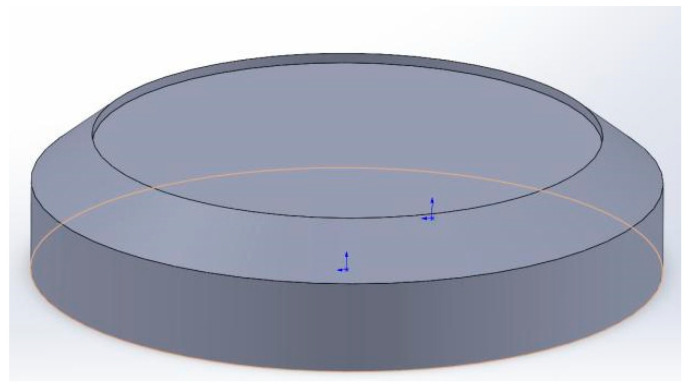
A flat plate modified into a concave groove structure diagram [54].

Cooksey et al. [54], from Australia, modified the disc by incorporating grooves to enhance both the morphology and the granulation conditions during the granulation process (Figure 11). CSIRO utilized a disc with a unique structural design, effectively suppressing the formation of slag wool. In the resultant blast furnace slag particles, approximately 90% were less than 1.5 mm in diameter.

**Figure 12:**
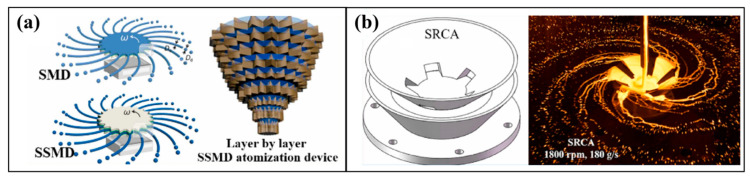
(**a**) The Spinning Multi-Disk (SMD) and Spinning “Sandwich” Multi-Tip Disk (SSMD) [58]. (**b**) Stacked Rotary Cup Atomizer (SRCA) [59].

Peng et al. [55,56,57] designed various structural forms of disc pelletizers and conducted extensive in-depth cold-state experiments and numerical simulations, laying a solid theoretical and practical foundation for hot-state experiments. However, systematic hot-state experimental results have not yet been published. Wang [58] developed the Spinning Multi-Disk (SMD) and the Spinning “Sandwich” Multi-Tip Disk (SSMD) (Figure 12a). Additionally, inspired by the transmission characteristics of pinecone seeds, a layer-by-layer solid particle diffusion design method that upgraded the droplet emission from two-dimensional to three-dimensional form was proposed. Subsequently, Tan et al. [59] proposed employing a Stacked Rotary Cup Atomizer (SRCA) under high slag flow rates to produce finer particles (Figure 12b).

### 3.2. Factors Influencing Granulation Effect in Numerical Simulation Studies

The dry centrifugal granulation technology for processing high-temperature molten slag involves a process characterized by high temperatures and high-speed rotation, imposing stringent requirements on the construction of the test rig for withstanding high temperatures and high strength. Additionally, the experimental conditions for high-temperature molten slag are harsh, resulting in high direct experimental costs and potential safety hazards. Consequently, alongside experimental research, numerical simulation of the blast furnace slag centrifugal granulation process has garnered extensive attention. Numerical simulation replaces some physical experiments through virtual simulation, enabling rapid verification of granulation effects under various working conditions. It also dynamically analyzes the physical mechanisms of slag fragmentation, cooling, and particle formation; optimizes process parameters; and aids scholars in further exploring the factors influencing centrifugal granulation.

#### 3.2.1. Simulation of Slag Spreading Process

Due to the close relationship between the thickness of the blast furnace slag liquid film and the size of granulated particles, Pan [60] pioneered a two-dimensional numerical simulation study on liquid film flow. This study considered the heat transfer between liquid slag, solidified slag, and the disk, investigating the effects of slag flow rate, initial temperature, and disk rotation speed on the liquid film’s thickness and temperature. Additionally, it predicted the thickness and temperature of the slag film prior to rupture. In 2019, Pan [61] utilized computational fluid dynamic (CFD) modeling techniques to examine the diffusion behavior of molten slag in a granulator through free surface flow simulation. The CFD model predicted the thickness of the liquid slag film on both the rotating disk and the cup’s top, revealing that the film thickness on the flat disk was less than that on the rotating cup.

**Figure 13:**
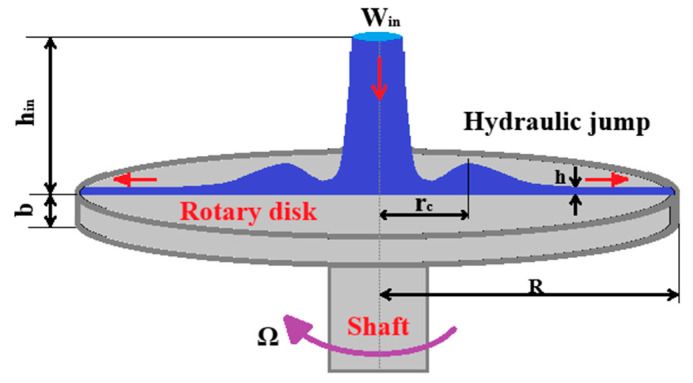
The film flow on the rotary disk (hydraulic jump phenomenon) [62].

Ling Xiang et al. [62] simplified and solved the momentum equation for blast furnace slag liquid film flow, thereby deepening the understanding of liquid film flow characteristics. Freystein and Wu Junjun [63,64] observed a fluctuating liquid film in their experiments, a feature not reflected in current two-dimensional numerical simulations. Consequently, Wang [65] developed a comprehensive three-dimensional model and discovered that a hydraulic jump phenomenon (Figure 13) occurs at high molten slag inlet velocities. It, however, does not impact granulation performance. Wang also delved into the formation mechanism and fluctuating behavior of the liquid film on the disk, analyzing the transformation processes of various flow patterns and their influencing factors.

The numerical simulation results from Wu et al. [44] demonstrate that the granulator’s structure significantly influences the liquid film thickness, with atypical structures effectively reducing this thickness. Chang Qingming and colleagues [66] simulated the dry centrifugal granulation (DCG) process of blast furnace slag using established physical and mathematical models, complemented by small-scale experimental validation. They systematically analyzed how slag temperature, viscosity, surface tension, and rotating disk speed affect granulation.

#### 3.2.2. Simulation of Slag Flight Process

The formation of slag droplets during flight is an exceptionally intricate process. Feng et al. [67] highlighted that this process is influenced by a multitude of factors, including centrifugal force, inertial force, surface tension, shear stress, drag force, and Rayleigh–Plateau instability. Mode conversion can be effectively achieved by manipulating physical and operational parameters. Furthermore, equations can be formulated based on experimental data and numerical simulation outcomes to precisely delineate modal transitions and variations in the number of liquid filaments.

Pan developed a transient three-dimensional model to qualitatively capture the pivotal characteristics of liquid filament formation and subsequent fragmentation. The diameter and depth of the RDA are crucial determinants of droplet formation. Consequently, Ling Xiang et al. undertook more comprehensive and meticulous three-dimensional numerical simulations, revealing that capillary waves are the primary driver of droplet formation. However, this study neglected the issue of heat exchange during flight. Qiu Yongjun et al. [68] employed the VOF method, coupled with solidification and melting models, for numerical simulation. Their findings indicated that smaller particle diameters and higher air velocities lead to shorter solidification times; the inlet air temperature has a negligible impact on the solidification process, whereas a higher initial slag temperature prolongs the duration of solidification. In multi-particle systems, particle spacing and distribution significantly influence heat exchange, while rotational speed can expedite the solidification process.

Du et al. [69] investigated the secondary fragmentation process of high-temperature blast furnace slag droplets through numerical simulation, utilizing the VOF method, RNG k-ε turbulence model, and solidification/melting model. They analyzed the bag-like fragmentation process of droplets under critical conditions. As droplet viscosity increases, the critical Weber number (We) also increases, and they derived the relationship between the critical We and Ohnesorge number (Oh) within the primary viscosity range of blast furnace slag.

Yang and colleagues [70] analyzed the flight, heat exchange, and solidification processes of blast furnace slag particle groups within the granulation chamber. The results indicated that neither the particle mass flow rate nor the initial temperature affects their trajectories. During solidification, a solid shell rapidly forms on the particle surface, yet the core remains incompletely cooled upon exiting the granulation chamber. Furthermore, an increase in air velocity thickens the solidified layer on the particle surface, whereas increasing the particle diameter or initial temperature decreases the thickness of this layer.

Liu Xiaoying and colleagues [71] conducted numerical studies on the phase change heat transfer characteristics during the air cooling of high-temperature molten blast furnace slag, developing one-dimensional temperature and enthalpy method models as well as a two-dimensional VOF-coupled solidification model. Their research revealed that the presence of a phase change temperature zone leads to the formation of a mushy region with coexisting solid and liquid phases. This extends the solidification time by 23% compared to conditions with a constant phase change temperature. The coupling effect of variable thermal conductivity and radiation heat transfer enhances the simulation’s realism, resulting in non-uniform solidification on the particle surface due to uneven flow fields, and a significant reduction in the solid phase growth rate after 80% solidification.

#### 3.2.3. Simulation of Process of Slag Impacting the Wall Surface

The phenomenon of droplets impacting solid surfaces is prevalent in both nature and industrial settings, where dynamic characteristics and heat transfer mechanisms significantly influence the overall performance of integrated systems. Xiang Yuhao and colleagues [72] conducted a numerical simulation to investigate the dynamic behavior and phase change heat transfer characteristics of molten blast furnace slag droplets impacting a wall. This study marked the first integration of the VOF method, a solidification–melting model, and a crystallization model. The research examined the impact of droplet diameter, impact velocity, and wall conditions on droplet spreading, heat transfer, and crystallization. Their findings indicated that larger droplet diameters increase the spreading area but decelerate the solidification rate. High-velocity impacts enhance heat transfer but also expand the spreading area. Additionally, the wall’s thermal conductivity significantly influences both the solidification time and the amount of crystallization.

**Figure 14:**
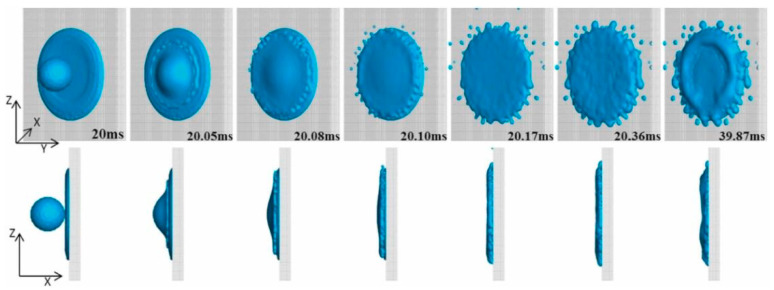
The morphology of the sequential impingement of two slag droplets [67].

Jahanshahi [73,74] discovered that when molten slag impacts a cold metal plate at varying angles, the contact time and maximum spreading length are influenced by the slag droplet’s surface roughness and initial diameter. The coupling effect between high-viscosity slag flow and heat transfer plays a more pivotal role in the evolution of slag droplets than the initial impact parameters. Feng [67] further explored the sequential impact of two slag particles of identical diameters and observed phenomena such as splashing and remelting (Figure 14).

### 3.3. Chapter Conclusion

The centrifugal granulation process of blast furnace slag is highly intricate, with scholars investigating it through both laboratory simulations and numerical simulations using commercial software. Research spanning from simulation media to high-temperature slag experiments has consistently shown that parameters such as granulator size, rotational speed, melt flow rate, and melt temperature significantly influence centrifugal granulation efficiency. Specifically, increasing rotational speed tends to reduce particle size, whereas decreasing rotational speed results in larger particles. An elevated melt flow rate leads to increased particle sizes but also enhances the uniformity of particle mass distribution. Additionally, the melt’s physical properties, including temperature, viscosity, and surface tension, critically impact the granulation process. For example, low MgO or high TiO_2_ content in blast furnace slag increases viscosity, thereby raising the proportion of slag formation and adversely affecting granulation efficiency. Despite the advantages demonstrated by various granulators in experimental settings, achieving industrial application necessitates further in-depth research.

## 4. Composite Granulation Process and Its Enhancements

Dry granulation technology for blast furnace slag is a crucial component in the green transformation of the steel industry. It stands to replace the traditional water-quenching process and achieve more efficient resource utilization and waste heat recovery of molten slag. Currently, mainstream dry granulation methods include centrifugal granulation and gas quenching. However, no single technology can adequately balance particle uniformity, waste heat recovery rate, and equipment durability. Centrifugal granulation produces small, highly spherical particles with high waste heat recovery rates and glass content, making it highly promising for resource utilization. However, given that it requires equipment with exceptional high-temperature resistance, is prone to particle agglomeration when handling large volumes of molten slag, and has somewhat limited cooling rates, its widespread application is constrained. Conversely, gas quenching demands substantial compressed gas, leading to high energy consumption, and results in particles with a broad size distribution and low sphericity. Additionally, its waste heat recovery system is relatively complex, thereby increasing operational costs and technical challenges.

Integrating different dry granulation methods through multi-technique coupling can fully exploit the synergistic benefits of each, thereby enhancing overall process efficiency. Yoshinaga first proposed a rotary disk centrifugal granulation process incorporating air holes. Mizuochi et al. [75] subsequently investigated the impact of gas flow rate on granulation effectiveness for this process, with experimental results showing that higher gas flow rates reduce particle size.

Zhu et al. [76,77] developed an experimental setup for a centrifugal–gas-quenching composite granulation fluidized bed waste heat recovery system, using rosin wax to simulate blast furnace slag. They examined the effects of rotating cup speed, liquid flow rate, and air injection flow rate on particle size, mass distribution, and fiber mass fraction. Their results indicated that higher rotational speeds and reduced liquid flow rates yield smaller particle sizes, although these conditions also lead to an increase in the fiber mass fraction. An increase in the air injection flow rate results in larger particle sizes and a higher fiber mass fraction. The study further explored the impact of air injection on granulation performance, highlighting the dominant role of cooling effects in the granulation process.

Japanese JFE Steel [78] has also investigated a composite process combining centrifugal granulation with air quenching. This process, which primarily involves the initial dispersion of molten slag through centrifugal force, followed by secondary fragmentation and enhanced cooling via high-speed airflow, significantly improves slag particle quality and waste heat recovery efficiency. Utilizing this centrifugal–air-quenching coupling process, slag particles achieve a specific surface area of 450 m^2^/kg, making them directly applicable in cement production and increasing the clinker substitution rate to 40%. These outcomes showcase the substantial advantages of this coupling technology in practical applications.

Beyond centrifugal and air-quenching composite technology, researchers have examined the dry granulation process for blast furnace slag using gas–water mixed quenching. Wan Xinyu et al. [79] conducted a 300 kg/h experiment to analyze the effects of air-quenching flow rate and atomized cooling water flow rate on granulation efficiency. Their findings revealed that an air-quenching flow rate of 40 Nm^3^/h and an atomized cooling water flow rate of 40 L/h resulted in highly spherical granulated particles, with over 85% of particle sizes below 5 mm and a glass content exceeding 95%. This process effectively reduces air consumption and enhances waste heat recovery efficiency, enabling the recovery of 0.22 tons of low-pressure steam per ton of slag, equivalent to 18.34 kg of standard coal.

In summary, the integration of various dry granulation methods through the combination of complementary strengths and process optimization significantly boosts the overall efficiency of blast furnace slag treatment. The combination of the centrifugal and air-quenching methods currently holds the most promise, as it effectively balances particle quality and waste heat recovery. Future research should prioritize verifying the stability and economic feasibility of this coupled process while also fostering policy and industrial chain collaboration. Through the integration of multiple technologies and system integration, the dry granulation coupling process is poised to become a pivotal technological pillar for the circular economy, and to significantly support the green transformation and sustainable development of the global steel industry.

**Figure 15:**
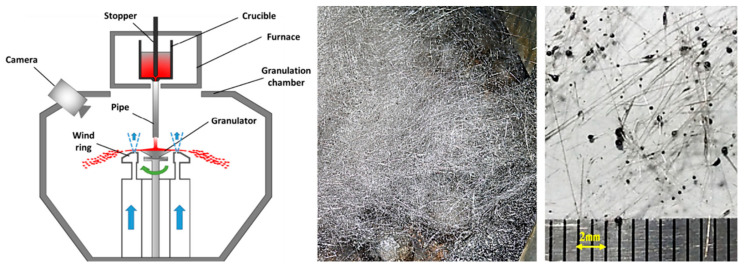
Centrifugal air blast granulation experimental setup and slag cotton obtained during the granulation process [80].

However, concerning the centrifugal–air-quenching composite technology of, Tan et al. [80] posit that airflow primarily enhances the granulation process by increasing shear forces and disturbance waves, with cooling effects playing a secondary role in particle size regulation. When the cooling effect surpasses the disturbance effect of the airflow, slag wool formation ensues (Figure 15). This issue of slag wool generation is also prevalent in standalone centrifugal granulation processes. Ma et al. [81] investigated the factors influencing the production of filamentous materials (slag wool) during the cup granulation of yellow phosphorus slag. Lower initial slag temperatures, reduced slag flow rates, larger cup diameters, and excessively high granulator speeds all contribute to an increase in the production of filamentous material. The formation of such material can be assessed by examining the relationship between the solidification time of the slag after it splits into liquid filaments and the time required for it to break into droplets. Filamentous material formation is indicated when the solidification time is shorter than the breaking time (t_solidification < t_break).

**Figure 16:**
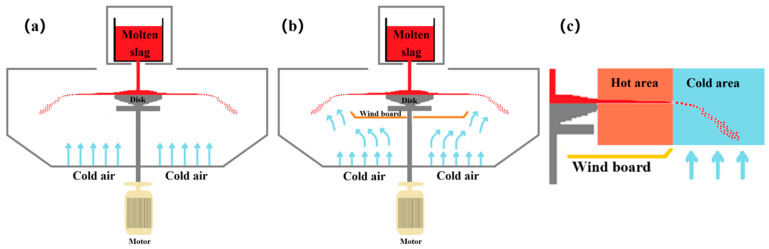
Centrifugal granulation equipment with windbreak and temperature zone diagram.

To address this contradiction, this study proposes an improved temperature zoning control method that aims to produce small-sized particles while suppressing slag wool formation. In the traditional process (Figure 16a), direct heat exchange between natural wind and the bottom of the granulation chamber causes two issues: Firstly, high-temperature molten slag ejected from the granulator’s edge as liquid filaments solidifies prematurely due to early cooling, leading to slag wool formation. Secondly, the granulation area requires adequate heat exchange for the rapid solidification of liquid droplets. To resolve these issues, this paper innovatively introduces a wind barrier device at the granulator’s bottom (Figure 16b), which optimizes the temperature field through airflow regulation (Figure 16c). The wind barrier prevents natural wind from directly impacting the liquid filament area. This reduces the convective heat exchange intensity, maintains a higher temperature environment, and extends the flow time of molten slag filaments, thereby effectively inhibiting slag wool generation. In the granulation area, where liquid filaments break into droplets, normal heat exchange between natural wind and high-temperature molten slag is preserved to ensure timely solidification into regular slag particles. The improved device successfully achieves the synergistic effect of “delayed solidification of liquid filaments and rapid granulation solidification”. This enhancement not only meets the industrial demand for small-sized slag particles but also reduces slag wool formation by delaying the solidification process, all while maintaining the device’s structural simplicity. It combines process optimization with economic feasibility, offering a novel technical approach for the resourceful treatment of metallurgical waste slag.

## 5. Conclusions

This article provides a comprehensive overview of the latest advancements in blast furnace slag centrifugal granulation technology. It delves into the current status and challenges of this technology from both experimental observation and computer simulation perspectives, and explores its future directions for development.

In this review related to experimental research, the significant impact of slag viscosity–temperature characteristics, a key physical property parameter, on the centrifugal granulation process has been clearly established. The nonlinear variation of this parameter directly determines the stability of the molten film and the droplet formation mechanism, thereby influencing the quality of the final slag particles. This insight offers a crucial theoretical foundation for optimizing the centrifugal granulation process, and can aid in the adjustment of process parameters based on physical properties to achieve a more ideal granulation effect.

Numerical simulation studies employing the CFD–DPM coupling model have successfully elucidated the synergistic mechanisms among various parameters, including the rotational speed of the cup, slag flow rate, and cooling air velocity. However, the field currently confronts technical bottlenecks in dynamically regulating thermodynamic parameters and controlling process stability, which hinder the further development and large-scale application of centrifugal granulation technology. Future research should delve into achieving precise dynamic regulation of thermodynamic parameters through a combination of model optimization and experimental validation, thereby enhancing the process’s stability and reliability.

The latest research introduces an innovative process combination—integrating centrifugal granulation with air-quenching cooling to create a multi-stage gradient cooling system—marking a significant breakthrough in centrifugal granulation technology. This hybrid approach not only substantially increases the cooling rate of slag particles but also optimizes their sphericity and particle size distribution, thereby boosting the energy efficiency conversion rate of the waste heat recovery system. This development not only offers a novel technical pathway for the resourceful treatment of metallurgical solid waste but also exhibits substantial theoretical value and application potential in the realms of circular economy and clean production. It is poised to drive technological innovation and sustainable development in the metallurgical solid waste treatment industry.

In response to the slag wool issue prevalent in both centrifugal granulation and centrifugal–gas-quenching composite processes, this paper innovatively proposes a new device designed to inhibit slag wool generation. This device achieves a synergistic effect through “liquid filament retardation and granulation rapid solidification”, effectively suppressing the formation of slag wool.

In summary, centrifugal granulation technology holds significant promise for the resourceful treatment of blast furnace slag. However, further progress necessitates collaborative efforts in both experimental research and numerical simulation to overcome existing technical bottlenecks. By refining the process system, this technology can achieve efficient and stable application in industrial production, thereby contributing more substantially to the green development of the metallurgical industry.

## Data Availability

Not applicable.
